# Magnitude of Diabetes Mellitus and Associated Factors Among HIV-Infected Individuals on Follow-Up Care at Kuyu General Hospital, North Shoa, Oromia, Ethiopia

**DOI:** 10.1155/jnme/7001308

**Published:** 2025-04-24

**Authors:** Sahilu Tesfaye Weyessa, Eyoel Berhan Mekonen, Tesfalem Teshome Tessema

**Affiliations:** ^1^Department of Public Health, St. Paul's Hospital Millennium Medical College, P.O. Box 1271, Addis Ababa, Ethiopia; ^2^Department of Medical Laboratory Science, Institute of Health Science, Wallaga University, Nekemte, Ethiopia

**Keywords:** ART, associated factors, diabetes mellitus magnitude, DM and HIV

## Abstract

**Background:** Antiretroviral therapy (ART) drugs improve life expectancy and reduce mortality. However, due to treatment-related metabolic complications, they are now developing comorbidities. In Ethiopia, there are a few reports of diabetes mellitus (DM)–human immunodeficiency virus (HIV) comorbidity. This study explores the magnitude of DM and associated factors among HIV-infected individuals on follow-up care at Kuyu General Hospital, Ethiopia.

**Materials and Methods:** A cross-sectional study design was conducted at Kuyu General Hospital from March 10, 2021–April, 2021. Adults with HIV-positive (aged ≥ 18 years) who were on ART were included. Systematic random sampling was used to select 294 HIV-positive adults who attended regular follow-up at the ART clinic. Descriptive analysis was conducted and reported in frequency and percentage. Both bivariable and multivariable analyses were computed. Variables with *p* < 0.25 in bivariable analysis were inserted into a multivariable logistic regression model to control possible confounders. The *p* value < 0.05 at a 95% confidence interval was considered as statistically significant.

**Results:** The age of the HIV-infected individuals enrolled ranged from 18 to 67 years with the mean age of 39.08 (SD = ±11.5) years. DM was detected in 21 (7.14%; 95% CI: 4.1–10.2) patients on medication whereas fasting plasma glucose between 111–125 mg/dL was 39 (13.3%; 95% CI: 9.5–17.3). The maximum (12.6%) of DM patients were aged 45 years and above. In the multivariable analysis, hypertension (AOR = 3.4, 95% CI: 1.1–10.8), elevated total cholesterol (AOR = 4.3, 95% CI: 1.2–15.6), aged 45 years and above (AOR = 3.9, 95% CI: 1.15–13.6), and duration of HIV (AOR = 4.7, 95% CI: 1.3–16.9) were significantly associated with DM.

**Conclusions:** In this study, the magnitude of DM among HIV-infected adults on ART follow-up was higher than the prevalence of DM in general populations. Older age, hypertension, increased total cholesterol, and duration of HIV were associated with a higher prevalence of DM. It is better for care providers assigned at ART clinics to detect DM, particularly after initiation of ART routinely, which may help to provide integrated care for comorbid patients.

## 1. Introduction

Globally, in 2020, people with human immunodeficiency virus (HIV) (PWH) were 37.7 million with 1.5 million new cases. Sub-Saharan Africa (SSA) remains with the highest disease burden [1], which contributes to two-thirds (76%) of the global total of people living with HIV (PLWH), and is the leading cause of morbidity and mortality [2, 3]. In Ethiopia, an estimated 690,000 PLWH and 65% of them were on highly active antiretroviral therapy (HAART) [4].

PLWH on combination with antiretroviral therapy (cART) had a higher prevalence (up to four times) and increased risk of diabetes when compared with HIV-uninfected individuals [5–8]. Advances in cART prolong the life expectancy of PLWH and reduce the mortality [1, 9–13]. However, the high prevalence of comorbidities associated with longer lives is now developing as novel challenges [9, 14, 15] due to the emergence of treatment-related metabolic complications [11, 16, 17]. Metabolic toxicities and elevation of circulating inflammatory cytokine levels caused by ART result in the releasing of circulating free fatty acids and lead to atherosclerosis that may impair insulin actions [9, 18–21].

Increasingly, nucleotide reverse transcriptase inhibitors (NRTIs) and protease inhibitors (PIs) have been associated with metabolic toxicities due to impairment of insulin action and also cause elevated serum triglycerides, total cholesterol (TC), and low-density lipoprotein cholesterol (LDL-C) [12, 22]. In the HIV-infected population, the glucose metabolic disorder ranged from 2% to 14% for diabetes globally [22–24]. In addition to clusters of differentiation 4 (CD4) count and duration and type of HAART [25], the increment in diabetes among people on ART is due to the same risk factors as the general population who develop type-2 diabetes mellitus (DM) [5, 26]. Increased age, high blood pressure, family history of diabetes [7, 17], unhealthy diets (high fat intake, sugars, and salt), alcohol consumption, and physical inactivity are among risk factors for noncommunicable diseases (NCDs) both in the HIV-infected population and the general population [3, 5, 16, 27]. HIV-DM comorbidities lead to a lowering of patients' quality of life as well as increased medical and societal costs, including other socioeconomic consequences [13, 25, 28, 29]. Although the burden of diabetes is high, the coverage, quality, and equity of services and access to prevention, care, and management for DM are a challenge in low-income countries where health system infrastructures provide underservices [16, 27, 28, 30]. This is supported by a study in Bahir Dar, Ethiopia, that suggests the health system is weak for diagnosing and providing service for NCDs among HIV services [27]. In addition, attention is given to PLWH to link to follow-up as early as possible to shorten the time between HIV diagnosis and ART initiation [8], but the emergence of this comorbidity was not considered. However, both HIV and DM have symptoms raised and declined gradually and require behavioral changes, prolonged self-treatment and persistent clinical follow-up and laboratory monitoring, care, and support [30], and thus, they need integrated care to minimize multiple visits. Although there were reports on the prevalence of DM in general populations in Ethiopia [29, 31, 32], evidence on the magnitude of DM among HIV-infected populations on follow-up care is still lacking. As a result, adequate information on identifying the magnitude of DM among HIV patients and assessing modifiable factors of this double burden leads to obtaining integrated and timely care for a better outcome. Thus, adequate studies enable us to develop policy and implementation of interventions which make us alert to prevent and control the risk of mortality and morbidity accelerated by comorbidity and to reduce medical care costs and loss of productivity.

Therefore, this study aimed to assess the magnitude of DM and its associated factors among HIV patients on follow-up care to fulfill the observed discrepancy in Kuyu General Hospital, Oromia, Ethiopia.

## 2. Methods and Materials

### 2.1. Study Setting

**Figure 1:**
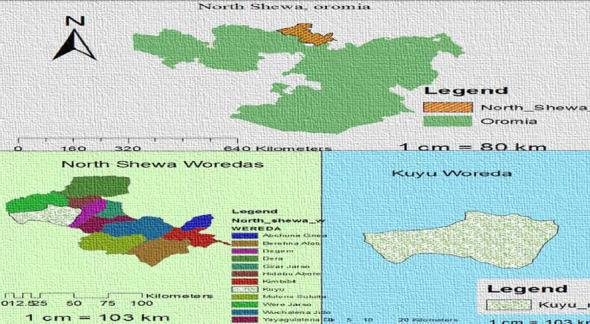
Map of study area, 2021.

The study was conducted at Kuyu General Hospital, which provides services for an estimated 245,000 people from Kuyu woreda and surrounding districts, including Gundo Meskel, Degem, Hidabu Abote, and Wore Jarso. The woreda in which the hospital is found is located 150 km to the North (North Shoa) from the capital Addis Ababa. Gerbe Guracha town, the administrative center of Kuyu woreda, is located at latitude and longitude 9048′N and 38,024′E and elevated between 2515 and 2547 m above sea level (Figure 1). Kuyu General Hospital was inaugurated in 2015 G C and has a total staff of 223 [33]. Kuyu Hospital also has different units, including the ART clinic and the laboratory department. Patients were voluntarily tested for HIV, and who were positive for HIV were counseled and linked to the ART clinic. Kuyu General Hospital provided service for ART patients, including CD-4 count, viral load, monitoring their organ function and, in case, complications occur, hospitalization of the patients.

### 2.2. Study Design and Period

We employed a cross-sectional study design to determine the magnitude of DM and associated factors among HIV-infected individuals on follow-up care at Kuyu General Hospital from March 10, 2021 to April, 2021.

### 2.3. Eligibility Criteria

Adults with HIV-positive whose age is 18 years and above were included in the study. However, hospitalized patients who were severely ill unable to respond to the interview, and pregnant women were excluded.

### 2.4. Study Variables

The outcome variable was the magnitude of DM. The sociodemographic characteristics including age, sex, residency, marital status, occupation, and educational status; genetic factors including family history of diabetes; behavioral factors including smoking, drinking alcohol, and physical activity; physical measurement factors including body mass index (BMI) and blood pressure; and clinical factors including duration of infection, duration on ART, CD4 level, TC, viral load, and fast plasma glucose after being on medication were independent variables.

### 2.5. Sampling and Sampling Procedure

The sample size was calculated using Epi Info Version 7.2.1 for a cross-sectional study. We employed a 95% confidence interval (CI), 80% power, 5.667 OR, 2.1% outcome in an exposed, and ratio of unexposed to exposed (1.5), based on a study conducted at Jimma University Specialized Hospital, Ethiopia [34], and finally, we obtained the total sample size including 10% contingency was 319 (Table 1).

We estimated the average number of HIV patients attending the ART clinic for follow-up in the previous 4 months. Patients were distributed uniformly each month and again each week. Depending on this estimation, we determined the number of patients who attended the ART clinic for the next two months, from March 10 to April 2021. The total HIV-positive adults who visited the ART clinic in the previous four months were 1192 and 596 for the past two months. Finally, we used systematic random sampling to select study participants (*K* = 596/319 = 2).

### 2.6. Data Collection

#### 2.6.1. Questionnaire

A pretested structured questionnaire adopted from the WHO STEPwise instrument and other literatures was developed and used to conduct face-to-face interviews to collect data on sociodemographic, medical and family history, and behavioral factors. The data were collected by five trained data collectors, including general practitioners, laboratory experts, and nurses who were able to speak and write the local language (Afan Oromo). The principal investigator provided training concerning the overall process of the study for those who collect the data. Clinical measures including duration of HIV infection and duration on ART were abstracted from patients' folders and database during the study period.

#### 2.6.2. Anthropometric Parameters

BMI was calculated as weight (kg) divided by the square of height in meters (kg/m^2^) and was interpreted as obesity, overweight, normal, and underweight when 30.0 and above, 25.0–29.9, 18.5–24.9, below 18.5, respectively [29, 35, 36].

We employed calibrated digital electronic devices to measure systolic blood pressure (SBP) and diastolic blood pressure (DBP). At least two readings were taken, and, in case readings differed by more than 5 mm Hg, we took additional readings until two or more closed or precise [37], and we incorporated the SBP for analysis.

### 2.7. Blood Collection

#### 2.7.1. Venous Blood Collection

After identifying the patients, verifying the patient was fasting, and then, we applied the tourniquet to collect approximately 5 mL of fasting venous blood specimen from each consenting study participant enrolled. To measure the serum glucose level, TC, and viral load, we collected the venous blood.

### 2.8. Quantitative Variables

To check the normality of the distribution, the Q–Q plot was used. For convenience of analysis, we categorized them based on WHO standards and using literatures. Fast blood glucose was grouped as DM when fasting plasma glucose (FPG) is greater than 126 mg/dL (FPG > 126 mg/dL) and prediabetes is fast blood sugar (FBS) 111–126 mg/dL. Descriptive analysis (mean, range, and standard) was conducted for age of the participants.

### 2.9. Data Quality Control

A structured questionnaire prepared for a face-to-face interview was translated to the local language and retranslated back to maintain consistency and was standardized by 5% pretesting on patients at Tulu Bolo General Hospital, Southwest Shewa, 2 weeks prior to the actual data collection. Before departure for data collection, 3 days' training was provided for data collectors and a supervisor concerning the objective of the study, data quality, and overall process of data collection. Based on the pretest, the questionnaire was reviewed and formatted. Throughout the entire collection process, close supervision was conducted by the principal investigators and supervisors. The collected data were checked for completeness.

#### 2.9.1. Quality Control for Blood Samples

Patients were oriented to be in the fasting state prior to the sample collection. We also oriented them to abstain from alcohol consumption for 24 h and, in the morning, neither drink caffeine-containing beverages nor smoke cigarettes prior blood sampling. Blood was drawn in the morning in a fasting state.

### 2.10. Statistical Methods

#### 2.10.1. Data Processing and Analysis

Consistency and cleanness of data were checked and entered into Epi Info Version 7.2.1 and exported to Statistical Package for Social Sciences (SPSS) Version 25 for further analysis. Descriptive analysis such as sociodemographic characteristics, other variables, and prevalence of DM was conducted and finally presented by frequency and percentage. After checking all the assumptions of logistic regression, variables with *p* < 0.25 in the bivariable analysis were inserted into a multivariable logistic regression model to control confounding factors. The *p* value < 0.05 at a 95% CI was considered as statistically significant.

### 2.11. Ethical Approval and Informed Consent

The Institutional Review Board (IRB) of the Saint Paul Hospital Millennium Medical College gave ethical approval for this study with approval number PM 23/513 on March 3, 2021. From Kuyu General Hospital and its respective departments (ART clinic and laboratory department), we also received a research permit to carry out the study. A written informed consent was obtained from each participant prior to enrolling to the study. The participants were clearly explained to provide information that would improve the care for diabetic patients.

### 2.12. Operational Definitions

#### 2.12.1. Blood Pressure is Categorized as

1. Normal blood pressure is when the SBP < 120 mmHg and DBP < 80 mm Hg.
2. Hypertension is when the pressure in the blood vessels is 140/90 mmHg or higher [38].

#### 2.12.2. Alcohol Consumer

Alcohol consumers include participants who consume any alcoholic drinks at least twice weekly [7].

#### 2.12.3. Physical Activities Are Classified as

1. High physical activities are vigorous activity for at least 3 days per week each lasting 30 min or more causes large increases in breathing or heart rate such as carrying or lifting heavy loads and digging or construction work.
2. Moderate physical activities are vigorous activity for 3 days per week each lasting 20 min or 5 days of walking for at least 30 min per day, which causes small increases in breathing or heart rate, such as brisk walking or carrying light loads.
3. Low physical activities are any activity less than those categories mentioned as high or moderate [27].

## 3. Result

### 3.1. Sociodemographic Characteristics of the Participants

A total of 294 HIV-infected adults on ART follow-up care who met the inclusion criteria were approached with a response rate of 92.2%, and 25 (11 pregnant and 14 adults in critical condition) were excluded. The age of 294 (217 females and 77 males) PLWH included in the study was ranged from 18 to 67 years with a mean age of 39.08 (SD = ±11.5) years. The mean age of females and males was 38.2 (SD = ±10.6) and 41 (SD = ±13.5), respectively. Out of 294 HIV-infected adults, 217 (73.8%) were females and 199 (67.7%) were urban dwellers. Among all participants, 145 (49.3%), 160 (54.4%), and 179 (50.9%) were married, had no formal education, and engaged in private occupation, respectively (Table 2).

### 3.2. Behavioral and Clinical Characteristics of the Participants

While analyzing the behavioral and clinical characteristics of the study participants, 8 (2.7%) and 3 (1%) consumed any alcohol and tobacco products, respectively. The majority, 181 (61.6%), had normal BMI. The clinical data showed that about 177 (60.2%), 278 (94.6%), and 268 (91.2%) were living with HIV for more than 5 years and had CD4+ T-lymphocytes less than 200 cells/mm^3^, and the most recent viral load was higher than 200 copies/mL, respectively (Table 3).

### 3.3. Magnitude of DM

Among 294 HIV-infected individuals, the prevalence of DM was 21 (7.14%; 95% CI: 4.1–10.2) whereas FPG between 111 and125 mg/dL was detected among 39 (13.3%; 95% CI: 9.5–17.3) adults attending ART follow-up care. Of the total DM patients, 13 (62%) knew their status before the study was conducted, and 8 (38%) were newly diagnosed. The magnitude among females was 5.5% (12/217) and 11.7% (9/77) among males. The maximum (12/95, 12.6%) of those having DM were greater than 45 years, followed by 18–34 (5/93, 4.9%) years age (Table 4).

### 3.4. Factors Associated With DM Among HIV Patients on Follow-Up

#### 3.4.1. Bivariable Analysis of Factors Associated With DM

From the sociodemographic characteristics' bivariable analysis, we obtained the following three factors: including sex, age group, and residency area. Besides this, the behavioral and clinical characteristics of the participants were also computed by bivariable analysis and also obtained four variables. Physical activity, hypertension, and TC in the blood and duration of HIV (> 5 years) were associated with DM at *p* < 0.25 (Table 4).

#### 3.4.2. Multivariable Analysis to Identify DM-Associated Factors

Variables independently associated with DM among HIV-infected adults were analyzed. Then, the overall factors associated with DM were identified after adjusting for possible confounders by the multivariable analysis. Finally, four variables statistically associated with DM, including high blood pressure (AOR = 3.4, 95% CI: 1.1–10.8), increased TC (AOR = 4.3, 95% CI: 1.2–15.6), aged 45 years and above (AOR = 3.9, 95% CI: 1.15–13.5), and duration of HIV (> 5 years) (AOR = 4.7, 95% CI: 1.3–16.9), were identified. Therefore, the prevalence odds ratio (POR) of having DM among hypertensive patients increases by a factor of 3.4 as compared to who had no hypertension (AOR = 3.4, 95% CI: 1.1–10.8, *p* < 0.04), and similarly, compared to whose TC ≤ 200 mg/dL, those who had TC > 200 mg/dL had higher prevalence ratio of having DM (AOR = 4.3, 95% CI: 1.2–15.6, *p* < 0.03). In addition, the prevalence ratio of having DM among age group greater than 45 years increases by a factor of 3.9 as compared to the age group between 18 and 34 years (AOR = 3.9, 95% CI: 1.15–13.5, *p* < 0.03) (Table 5).

## 4. Discussion

This study sought to assess the magnitude of DM and to determine the associated factors with DM among HIV-infected patients on follow-up care at the ART clinic of Kuyu General Hospital. The overall magnitude of DM among HIV-infected adults was 7.14%, in which males and older individuals aged 45 years and above were more affected. Factors associated with DM were older age, hypertension, and TC in the blood and prolonged duration of using ART among HIV-infected patients.

The findings of our study revealed that the magnitude of DM was 7.14% among HIV-infected individuals. The obtained magnitude in our study was higher than the prevalence in adults aged 20–79 years (4.8%) in Ethiopia in 2014 [29], which is supported by studies conducted in the Cameroon [17], Zambia [23], Zimbabwe [25], and Northwest Ethiopia [34], in which the prevalence was higher among HIV patients as compared to general population. This may be due to HIV-/AIDS-infected individuals being more likely to develop the DM compared to the general population, due to the effects of the prolonged HAART and ongoing inflammation due to chronic HIV infection [18].

Metabolic toxicities induced by older NRTIs and first-generation ART drugs may lead to increment of circulating free fatty acids accumulation [9], which may reduce action of insulin hormone in the liver and skeletal muscle [20]. In addition, in most HIV-infected adults on ART, the circulated free fatty acids released due to chronic gastrointestinal enteropathy, impaired mucosal defenses, and elevation of circulating inflammatory cytokine levels or immune system reaction may impair the action of insulin and lead to dysglycemia and DM [20–22, 25]. Moreover, PIs inhibit the effect of protein glucose transporter type 4 (GLUT4) and reduce the sensitivity of insulin [9]. Although a higher prevalence is observed among HIV patients than in the general population, the finding from our study was slightly similar with the studies conducted in Harar, Ethiopia [35], Jimma, Ethiopia [34], and Cambodia [39], where diabetes ranged from 6.4% to 8%.

In contrast, the prevalence rates of DM ranged from 0.5% to 5.8% reported from recently conducted studies [11, 21, 23]. However, evidences show that higher prevalence rate among HIV-infected individuals ranging up to 26% was reported from studies conducted in Africa [7, 17, 21, 22]. The variation in magnitude may be due to lifestyle, sociodemographic and use of older generation ART agents, age distribution of PLWH, and health inequality. Furthermore, the observed higher prevalence in high-income countries (HICs) might be due to more case detection using improved screening and diagnostic services. Evidence documented that the Ethiopian health system set up is poor at screening and diagnosing DM among HIV-positive people and providing integrated services for comorbidities [27].

In previous studies, the risk of DM increased as age increases among patients with HIV, including those on ART [7, 21, 27, 34]. A rise in the prevalence of metabolic disorders such as alterations in glucose tolerance was observed among patients who were of older age [4, 11, 13, 27, 40, 41]. Similarly, in our study, individuals aged 45 years and above were more affected. This might be due to the effect of ART or physiological changes.

Several studies have reported an increased prevalence of DM among male HIV-infected individuals [11, 24, 42]. Consistently, in our study among HIV-infected, males were more affected. However, this study is not in agreement with a study conducted at Jimma University Specialized Hospital, Southwest Ethiopia, in which diabetes was most prevalent among females [34]. However, differences in the resistance of insulin between sexes, specifically the role of sex hormones, are still under investigation [13].

Access and growth of HAART may lead to an increase in metabolic dysfunctions, dyslipidemia [29], and hypercholesterolemia [34]. Consistent with previous studies, there was a strong association between TC and prevalent DM among HIV-infected individuals [29, 34, 35], and most of the DM patients among adult HIV-patients on follow-up had higher TC.

In patients living with HIV, both DM and hypertension are rising and are the leading risk factors for mortality [7, 27, 34]. DM is commonly associated with other comorbidities such as hypertension [4, 18, 43]. The magnitude of hypertension was significantly increased among HIV patients on ART and is also an abnormality related to ARTs [5]. Similarly, in our study, there was an association between diabetes and high blood pressure among HIV patients on ART follow-up.

Although HAART improves the life expectancy of HIV patients, prolonged duration of ART has been described as a risk factor for the development of comorbidities that may be due to prolonged survival and aging. In addition, a follow-up study suggested that patients who had a longer length of time of exposure to ART were at the highest risk of diabetes [9]. In our study, a longer duration of using ART drugs was associated with DM development, which is consistent with other studies conducted previously [7, 34, 41].

In previous studies, BMI may contribute to the magnitude of diabetes among HIV-infected adults [17, 24]. However, the finding from our study revealed that there is no association with BMI, which was concordant with a study conducted by Husain et al. [22].

The strengths of this study include that we tried to adhere to the study protocol and selected samples based on the inclusion requirements to maintain representativeness. The definitions of terms and variables were consistent with previous studies and existing guidelines, particularly WHO guidelines. We conducted double entry of data from the paper-based questionnaire into the electronic database (from Epi Info to SPSS) in order to minimize errors. Moreover, a more recent viral load was also assessed for HIV patients on follow-up. However, some variables such as hepatitis C virus (HCV) tests, high-density lipoprotein (HDL), and LDL were not included in this analysis, although they could be among the associated factors observed in HIV-infected adults.

## 5. Conclusion

The magnitude of DM was comparably higher among HIV-infected individuals on ART follow-up care in Kuyu General Hospital. HIV-infected adults on ART follow-up had a higher prevalence at older age and higher among males. Older age, having high blood pressure, TC and prolonged duration of using ART were significantly associated with a higher prevalence. Therefore, based on our study findings and recent literature concerning the prevalence of DM among HIV-infected individuals, there are several important implications. Hence, it is better care providers assigned at ART clinic to conduct DM screening for all HIV-infected individuals on follow-up in their routine care.

## Figures and Tables

**Table 1:** Sample size calculation, Kuyu General Hospital, North Shoa, Oromia, Ethiopia, 2021.

S/N	Variables	Ratio (unexposed: exposed)	% outcome in unexposed	OR	Calculated sample size with CI 95%, power 80%
1	Age	4.23	21.8	3.51	162
2	Duration of using HAART	0.503	2.1	26.9	59
**3**	**LDL-C ** ^**∗**^	**1.503**	**2.1**	**5.667**	**290**
4	Family history of diabetes	8.7	28.6	6.64	121
5	Hypertension	4.85	20.3	3.45	185

^∗^indicates the largest sample size and written in bold.

**Table 2:** Sociodemographic characteristics of HIV-infected patients attending Kuyu General Hospital, North Shoa, Oromia, Ethiopia, 2021 (*n* = 294).

Characteristics	Number of participants	Percent
Age group (years)		
18–34	103	35
35–44	96	32.7
≥ 45	95	32.3
Sex		
Female	217	73.8
Male	77	26.2
Residency		
Urban	199	67.7
Rural	95	32.3
Educational status		
No education	160	54.4
Primary	83	28.2
Secondary	36	12.2
Degree/college	15	5.1
Marital status		
Single	15	5.1
Married	145	49.3
Divorced	78	26.5
Widowed	56	19
Occupation		
Self-employed	235	79.9
Employed	18	6.1
Unemployed	41	13.9

**Table 3:** Behavioral and clinical characteristics of the HIV-infected patients attending Kuyu General Hospital, North Shoa, Oromia, Ethiopia, 2021 (*n* = 294).

Characteristics	Number of participants	Percent
Consumption of alcohol		
Yes	8	2.7
No	286	97.3
Smoking habit		
Yes	3	1
No	291	99
Physical activity		
High	87	29.9
Moderate-intensive	154	52.4
Low	53	18
Hypertension		
Yes	40	13.6
No	254	86.4
BMI		
< 18.5	104	35.4
18.5–24.9	181	61.6
≥ 25	9	3.1
Family history of DM		
Yes	15	5.1
No	279	94.9
Total cholesterol (mg/dL)		
≤ 200	268	91.2
> 200	26	8.8
CD4 count (cell/mm^3^)		
≤ 200 cell/mm^3^	16	5.4
> 200 cell/mm^3^	278	94.6
Duration of HIV (years)		
≤ 5 years	117	39.8
> 5 years	177	60.2
Duration on ART (years)		
≤ 5 years	112	38.1
> 5 years	182	61.9
Most recent viral load (copies/mL)		
≤ 200	268	91.2
> 200	26	8.8

**Table 4:** Bivariable analysis for factors associated with DM among HIV-infected patients attending Kuyu general hospital, North Shoa, Oromia, Ethiopia, 2021 (*n* = 294).

Characteristics	Diabetes *n* (%)	No diabetes *n* (%)	COR (95% CI)	*p* value
Age group (years)				
18–34	5 (4.9)	98 (95.1)	1.00	
35–44	4 (4.2)	92 (95.8)	0.85 (0.22–3.27)	0.81
≥ 45	12 (12.6)	83 (87.4)	2.9 (1.0–8.7)	0.052^∗^
Sex				
Female	12 (5.5)	205 (94.5)	1.00	
Male	9 (11.7)	68 (88.3)	2.26 (0.9–5.6)	0.08^∗^
Residency				
Urban	12 (6)	187 (94)	2.1 (0.7–6.5)	0.19^∗^
Rural	9 (9.5)	86 (90.5)	1.00	
Educational status				
No education	7 (4.3)	153 (95.7)	0.46 (0.07–5.6)	0.69
Primary	7 (8.4)	76 (91.6)	1.3 (0.14–11)	0.82
Secondary	6 (16.7)	30 (83.3)	2.8 (0.3–25.5)	0.36
Degree	1 (6.7)	14 (93.3)	1.00	
Marital status				
Single/divorced/widowed	10 (6.7)	139 (93.3)	0.9 (0.36–2.1)	0.77
Married	11 (7.6)	134 (92.4)	1.00	
Occupation				
Employed	2 (11.1)	16 (88.9)	1.00	
Self-employed	14 (6)	221 (94)	0.5 (0.1–2.4)	0.39
Unemployed	5 (12.2)	36 (87.8)	1.1 (0.2–6.3)	091
Alcohol consumption				
Yes	1 (12.5)	7 (87.5)	1.9 (0.28–16)	0.55
No	20 (7)	266 (93)	1.00	
Physical activity				
High	4 (4.6)	83 (95.4)	1.00	
Moderate	16 (10.4)	138 (89.6)	2.4 (0.78–7.4)	0.13^∗^
Low	1 (1.8)	52 (98.2)	0.4 (0.04–3.7)	0.41
BMI				
≤ 25	20 (7)	265 (93)	1.00	
> 25	1 (11)	8 (89)	1.6 (0.9–13.9)	0.64
Hypertension				
Yes	6 (15)	34 (85)	2.8 (1.02–7.4)	0.005^∗^
No	15 (5.9)	239 (94.1)	1.00	
Family history of diabetes				
Yes	2 (13.3)	13 (86.7)	2.1 (0.44–10.1)	0.35
No	19 (6.8)	260 (93.2)	1.00	
CD-4 count (cells/mm^3^)				
≤ 200	1 (6.2)	15 (93.8)	1.8 (0.4–8.5)	0.45
> 200	20 (7.2)	258 (92.8)	1.00	
Total cholesterol (mg/dL)				
≤ 200	16 (6)	252 (94)	1.00	
> 200	5 (19.2)	21 (80.8)	3.8 (1.3–11.2)	0.02^∗^
Duration of HIV (years)				
≤ 5	4 (3.4)	113 (96.6)	1.00	
> 5	17 (9.6)	160 (90.4)	3 (0.98–9.1)	0.05^∗^
Duration on ART				
≤ 5	6 (5)	106 (95)	1.00	
> 5	15 (8.3)	167 (61.9)	1.6 (0.6–4.2)	36
Most recent viral load (copies/mL)				
≤ 200	19 (7.1)	249 (92.9)	1.00	
> 200	2 (7.7)	24 (92.3)	1.8 (0.5–6.6)	0.37

*Note:* HAART = highly active antiretroviral therapy.

Abbreviations: CI = confidence interval, COR = crude odds ratio.

^∗^Variables that are significant at *p* < 0.25.

**Table 5:** Multiple logistic regression for factors associated with DM among HIV-infected patients attending Kuyu General Hospital, North Shoa, Oromia, Ethiopia, 2021 (*n* = 294).

Characteristics	COR (95% CI)	*p* value	AOR (95% CI)	*p* value
Age group (years)				
18–34	1.00			
35–44	0.85 (0.22–3.27)	0.81	0.09 (0.2–3.8)	0.89
≥ 45	2.9 (1.0–8.7)	0.052	**3.9 (1.15–13.6)**	0.03^a^
Sex				
Female	1.00			
Male	2.26 (0.9–5.6)	0.08	1.7 (0.6–4.9)	0.3
Residency				
Urban	2.1 (0.7–6.5)	0.19	3.2 (0.92–11.2)	0.075
Rural	1.00			
Physical activity				
High	1.00			
Moderate	2.4 (0.78–7.4)	0.41	2.1 (0.6–7)	0.23
Low	0.4 (−3.7)	0.13	0.3 (0.03–3.2)	0.33
Hypertension				
Yes	2.8 (1.02–7.4)	0.005	**3.4 (1.1–10.8)**	0.04^a^
No	1.00			
Total cholesterol (mg/dL)				
≤ 200	1.00			
> 200	3.8 (1.3–11.2)	0.02	**4.3 (1.2–15.6)**	0.03^a^
HIV duration (years)				
≤ 5	1.00			
> 5	3 (0.98–9.1)	0.05	**4.7 (1.3–16.9)**	0.024^a^

*Note:* Confidence intervals that do not overlap 1 are shown in bold.

Abbreviations: AOR = adjusted odds ratio, CI = confidence interval, COR = crude odds ratio.

^a^ *p* < 0.05, statistically significant association.

## Data Availability

The main part of the data generated or analyzed during this study was included in this manuscript.
